# A Novel Machine Learning Model and a Web Portal for Predicting the Human Skin Sensitization Effects of Chemical Agents

**DOI:** 10.3390/toxics12110803

**Published:** 2024-11-07

**Authors:** Ricardo Scheufen Tieghi, José Teófilo Moreira-Filho, Holli-Joi Martin, James Wellnitz, Miguel Canamary Otoch, Marielle Rath, Alexander Tropsha, Eugene N. Muratov, Nicole Kleinstreuer

**Affiliations:** 1 National Toxicology Program Interagency Center for Evaluation of Alternative Toxicological Methods (NICEATM), Division of Translational Toxicology, National Institute of Environmental Health Sciences, Research Triangle Park, Durham, NC 27711, USA; ricat@unc.edu (R.S.T.); teofilo.moreirafilho@nih.gov (J.T.M.-F.); 2 UNC Eshelman School of Pharmacy, University of North Carolina, Chapel Hill, NC 27514, USA; holli27@email.unc.edu (H.-J.M.); jwellnitz@unc.edu (J.W.); motoch@email.unc.edu (M.C.O.); mrath@email.unc.edu (M.R.); 3 Predictive LLC, (A.T.), Chapel Hill, NC 27514, USA

**Keywords:** skin sensitization, computational toxicology, QSAR, cheminformatics, NAMs

## Abstract

Skin sensitization is a significant concern for chemical safety assessments. Traditional animal assays often fail to predict human responses accurately, and ethical constraints limit the collection of human data, necessitating a need for reliable in silico models of skin sensitization prediction. This study introduces HuSSPred, an in silico tool based on the Human Predictive Patch Test (HPPT). HuSSPred aims to enhance the reliability of predicting human skin sensitization effects for chemical agents to support their regulatory assessment. We have curated an extensive HPPT database and performed chemical space analysis and grouping. Binary and multiclass QSAR models were developed with Bayesian hyperparameter optimization. Model performance was evaluated via five-fold cross-validation. We performed model validation with reference data from the Defined Approaches for Skin Sensitization (DASS) app. HuSSPred models demonstrated strong predictive performance with CCR ranging from 55 to 88%, sensitivity between 48 and 89%, and specificity between 37 and 92%. The positive predictive value (PPV) ranged from 84 to 97%, versus negative predictive value (NPV) from 22 to 65%, and coverage was between 75 and 93%. Our models exhibited comparable or improved performance compared to existing tools, and the external validation showed the high accuracy and sensitivity of the developed models. HuSSPred provides a reliable, open-access, and ethical alternative to traditional testing for skin sensitization. Its high accuracy and reasonable coverage make it a valuable resource for regulatory assessments, aligning with the 3Rs principles. The publicly accessible HuSSPred web tool offers a user-friendly interface for predicting skin sensitization based on chemical structure.

## 1. Introduction

Skin sensitization testing is employed to determine the potential for a substance to cause allergic contact dermatitis in susceptible individuals [1]. United States and international regulatory authorities require or recommend that chemical manufacturers conduct tests that assess skin sensitization hazards [2]. The most common tests are the human repeat insult patch test, human maximization test, murine local lymph node assay (OECD TG 429 [3]), guinea pig maximization test (OECD TG 406 [4]), and Buehler test (OECD TG 406 [4]), although several non-animal tests now exist (OECD TG 442C; OECD TG 442D; OECD TG 442E; OECD TG 497) [5,6,7,8]. Despite the widespread use of animal tests, it has been shown that animal-based assay outcomes do not always equate with human responses [9] and that animal models are less reproducible than other alternative methods [10].

Human data provide the most accurate assessment of sensitization potential, but ethical considerations preclude the intentional induction of sensitization in human subjects, resulting in limited availability of such data. Despite this sparsity, the human repeat insult patch test (HRIPT) and the human maximization test (HMT) provide sufficient information on the potential of substances to cause allergic contact dermatitis. These assays include results detailing the dose per skin area (DSA) causing induction of sensitization, the number of individuals sensitized, and other parameters reflecting the sensitization potency from test substances. Reliable in silico models leveraging human assay data for skin sensitization predictions could support improved regulatory assessments.

Computational methods have become increasingly vital in toxicology in light of the three Rs (reduce, refine, and replace) of animal testing. In silico approaches are cost-effective, safe, and ethical alternatives to traditional animal and human testing. One computational approach explored here is machine learning (ML), in which algorithms enable systems to learn and improve from experience without being explicitly programmed [11]. Various computational models for skin sensitization already exist (Table 1).

**Figure 1:**
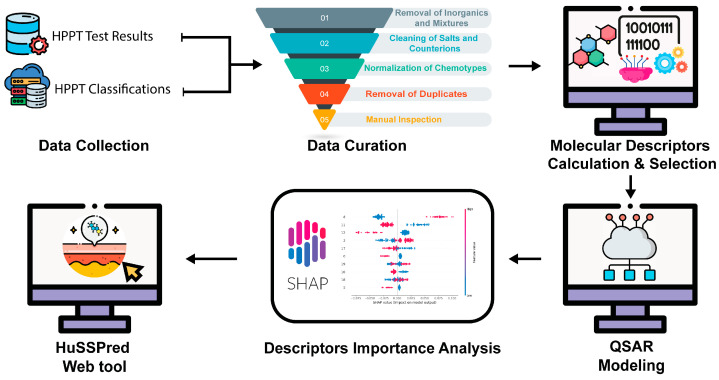
General study design of HuSSPred. Experimental data were collected from the HPPT test results and combined with the HPPT GHS Classifications database. All entries were carefully curated following best practices in the field. Molecular descriptors were calculated and selected to build skin sensitization QSAR models. SHAP analysis was performed to enhance the model’s interpretability. The best-performing QSAR models were deployed as a web tool, HuSSPred, available at https://husspred.mml.unc.edu/, accessed on 18 October 2024.

The purpose of this work is to present HuSSPred, an in silico human predictive patch test (HPPT)-based tool for Human Skin Sensitization Prediction from the human repeat insult patch test (HRIPT) and the human maximization test (HMT) (Figure 1). This study continues the series of our previous works on skin sensitization [20,21,22], extending the strategy that we developed earlier [23] in modeling human data. Using an extensively curated HPPT database, we developed QSAR models using random forest (RF), LightGBM, and support vector machine (SVM) algorithms with Bayesian hyperparameter optimization. Our models exhibit comparable or improved performance versus existing tools. We conclude that HuSSPred is one of the first tools built strictly with human data and validated with skin sensitization-defined approaches (DA) [24,25]. This tool provides a reliable, cost-effective, and ethical alternative to traditional testing for skin sensitization. Its accuracy and reasonable coverage make it a valuable resource for regulatory assessments, aligning with the three Rs principles. Furthermore, the HuSSPred web tool (https://husspred.mml.unc.edu/, accessed on 18 October 2024) is publicly accessible and offers a user-friendly interface for predicting skin sensitization based on chemical structure.

## 2. Materials and Methods

### 2.1. Data Collection and Curation

We collected chemical data from the NICEATM human predictive patch test (HPPT) [26,27] and the HPPT Classifications according to the Globally Harmonized System of Classification and Labelling of Chemicals (GHS) [28]. The HPPT database contains 2277 entries for 1366 unique chemicals. Using Chemical Abstracts Service Registry Number (CASRN), the HPPT data were harmonized with the Dose per Skin Area (DSA05) classifications database, resulting in 2014 entries. The data sets were then biologically and chemically curated according to the best practices in the field [29,30,31]. As for chemical curation, we removed mixtures, inorganics, and large organic compounds, removed counterions, cleaned and neutralized salts, and normalized chemotypes using the ChemAxon Standardizer software [32]. We followed one of the two appropriate procedures for handling duplicates: (i) if the outcomes of all duplicates were concordant (from a hazard perspective), one record was kept with the respective outcome; (ii) if any outcomes disagreed, they were removed. The HPPT data contained four different weight-of-evidence approaches with skin sensitization outcomes: the median location-like parameter (MLLP), median sensitization potency (MSPE), weight of evidence (WoE), and overall weight of evidence (WES). Most curation steps were performed through Konstanz Information Miner (KNIME) [33,34,35].

#### 2.1.1. Binary Data Curation

After combining the two data sets, we kept only binary labeled outcomes (sensitizers (1) and non-sensitizers (NCs)). Entries with missing outcomes were removed. Then, the dataset-specific curation began for each weight-of-evidence approach, through which we removed outcome entries with the label “unavailable” for MLLP, MSPE, WoE, and WES; this process was performed separately, resulting in 4 independent datasets. Compounds for which the SMILES could not be retrieved were also removed. We labeled all sensitizers as Class 1 and non-sensitizers as Class 0. The resulting data sets were exported as SD files into Python for model development (available in Supplemental File S1).

#### 2.1.2. Multiclass Data Curation

The collected data contained multiclass GHS classifications for skin sensitization potency. The outcome labels were mapped to numbers for model building, where NC (non-sensitizer), 1B (weak sensitizer), and 1A (strong sensitizer) were mapped to classes 0, 1, and 2, respectively. We removed compounds with missing outcomes and SMILES from each weight-of-evidence approach. The four multiclass data sets are available in Supplemental File S1.

#### 2.1.3. Continuous Data Curation

The data set entries also included continuous values for the dose per skin area (DSA), dose per skin area for potential sensitization of 5% of the tested population (DSA05), and dose per skin area for potential sensitization of at least 1 individual (DSA01+). We excluded compounds with missing outcomes, applied our standard chemical curation workflow, and calculated the median for compounds with multiple entries. We applied a logarithmic transformation to the DSA values.

### 2.2. Classification QSAR Modeling

#### 2.2.1. Calculation of Molecular Fingerprints

The RDKit package [36] was used to calculate extended-connectivity fingerprints with a diameter of 4 (ECFP4) and 2048 bits [37]. Molecular ACCess System (MACCS, version 3) Fingerprints [36], Mordred [38], and Saagar descriptors [39] were also used for model development.

#### 2.2.2. Model Development and Performance Assessment

Models were developed with either RF, LightGBM, or SVM algorithms. QSAR models were developed and validated according to the best practices in cheminformatics [40]. In the RF algorithm, trees were decorrelated via bootstrapping with replacement; in LightGBM, a gradient-boosting algorithm optimizes model performance through a leaf-wise tree construction approach. Models using LightGBM [41] and RF [42] were implemented through Scikit-learn, v.1.4.0 [43]. The SVM algorithm finds the optimal hyperplane that maximizes the margin between distinct classes in a multidimensional feature space, thereby enabling robust data point classification. These three ML algorithms were also utilized in similar studies, and they are dominant in the field based on a balance between computational efficiency, predictivity, and interpretability [44].

For binarized models (non-sensitizers vs. sensitizers), the following statistical metrics were used to assess the performance of the classification models (Equations (1)–(5)):

Sensitivity (SE):

(1) $$SE=\frac{N_{TP}}{N_{TP}+N_{FN}},$$

Specificity (SP):

(2) $$SP=\frac{N_{TN}}{N_{TN}+N_{FP}}\;,$$

Correct classification rate (CCR):

(3) $$CCR=\frac{SE+SP}{2},$$

Positive predictive value (PPV):

(4) $$PPV=\frac{N_{TP}}{N_{TP}+N_{FP}},$$

Negative predictive value (NPV):

(5) $$NPV=\frac{N_{TN}}{N_{TN}+N_{FN}}$$

N represents the number of compounds, *N_TP_* and *N_TN_* represent the number of true positives and negatives, respectively, and *N_FP_* and *N_FN_* represent the number of false positives and false negatives, respectively.

For binary classification models, compounds labeled as skin sensitizers were classified as positive (Class 1), and non-sensitizing compounds were classified as negative (Class 0).

For multiclass classification, compounds are labeled as non-sensitizers (Class 0), weak sensitizers (Class 1), or strong sensitizers (Class 2). The one-vs.-rest approach was used when calculating metrics for multiclass models. Metrics (such as SP and NPV) were computed for each class separately and then averaged to obtain an overall score. Classes were weighted equally (macro-average) during calculations for the average

#### 2.2.3. Hyperparameter Optimization

Since the performance of a model can be closely linked to the hyperparameters used during development, the models were optimized using a Bayesian approach implemented through Optuna [45]. Optuna uses a framework to identify ideal hyperparameters for a given set of descriptors and ML algorithms. The best hyper-parameters were then used to fine-tune the models using the entire training set of compounds and tested during the 5-fold cross-validation step.

#### 2.2.4. Dimensionality Reduction and Feature Selection

Dimensionality reduction was performed by applying a low-variance filter with a threshold of 0.01. Descriptors with low variance (threshold of 0.01) were filtered out to exclude non-informative data using Scikit-learn [43]. Subsequently, supervised feature selection was conducted using recursive feature elimination (RFE) to identify the most relevant molecular descriptors, thereby improving model performance and interpretability while reducing overfitting and training time [46].

#### 2.2.5. Normalization of Continuous Descriptors

We normalized the Mordred descriptors using min–max scaling to ensure consistent scale and improve model performance. The normalization was implemented via the “MinMaxScaler” class from the Scikit-Learn library [43], which scales each feature to a range [0–1]. The scaling process adjusts each descriptor’s value to be within the specified range, preserving the relationships among features while enhancing model training stability. Scaling was performed within each fold to prevent data leakage, ensuring that the scaling parameters were derived solely from the training data [47].

#### 2.2.6. Data Set Split and 5-Fold Cross-Validation

Five-fold external cross-validation was used [48]. For this, the data set was split into five equal parts, wherein one subset (20%) is used as the test set, and the remaining compounds (80%) compose the training set. This procedure was repeated five times, and each subset was used as the validation set exactly once. Models were built using the training set only, and compounds in the test set had to not be present in the training set. Alternative validation methods, such as 10-fold cross-validation and leave-one-out cross-validation, are available. We selected 5-fold cross-validation due to its established validity within the field, effectively balancing methodological rigor with computational efficiency [44].

#### 2.2.7. Threshold Moving

We tried the threshold-moving calibration of probability estimates to increase prediction confidence without losing data, i.e., without needing to balance the data. Binary QSAR models’ probability thresholds were adjusted using this approach, which was incorporated into Python [43,49]. Threshold moving was used to select the binary classification probability threshold for the model that produced the highest geometric mean values on these test sets. The geometric mean was chosen since it better assesses the performance of models when predicting imbalanced data [49,50,51,52]. Radar charts to represent model calibration were constructed using the Plotly package [53].

#### 2.2.8. Applicability Domain (AD)

Our group has previously shown the importance of the applicability domain of models when drawing conclusions from predictions derived from QSAR models. The AD must be stated for the given chemical space of predictive models to identify “reliable” and “unreliable” regions for predictions [49,54]. Thus, users should only consider the model’s predictions reliable if their predicted compounds fall within the model’s AD.

The “Applicability Domain” meta-node was used to assess the AD of our models. The KNIME meta-node uses Euclidean distances to measure the chemical similarity between a compound from the test set and its nearest neighbor in the training set. The prediction may be unreliable if the distance of a compound not present in the test set to its nearest neighbor is higher than an arbitrary parameter (Z = 0.5) that controls the significance level [55].

#### 2.2.9. Model Interpretation

Model interpretation was facilitated using machine learning interpretable packages such as Shapley additive explanations (SHAP) [56,57]. SHAP can be used to identify key features that impact model performance, improving the transparency and understanding of ML models [58].

Contribution maps [59,60] were generated from QSAR models to visualize atoms and fragments contributing to skin sensitization potential. The contribution maps used an approach in which an atom’s “weight” was considered a predicted probability difference obtained when bits in the fingerprints corresponding to the atom were removed. Then, the normalized weights were used to color atoms in a topography-like map in which green indicates the contribution to toxicity (i.e., predicted sensitization probability decreases when bits are removed) and red indicates a negative contribution to toxicity (i.e., predicted sensitization probability increases when bits are removed) [60].

#### 2.2.10. Model Implementation—HuSSPred Web Tool

The QSAR models developed in this study were implemented as a web application, HuSSPred, which runs on an Ubuntu server. The HuSSPred application is encoded using Flask [61], uWSGI [62], Nginx [63], Python 3.11.4 [64], RDKit [36], Scikit-learn [43], and JavaScript [65]. HuSSPred also includes the JSME molecule editor [66], written in JavaScript and supported by most popular web browsers. The server takes input chemicals and produces skin sensitization predictions based on chemical structure for the user.

### 2.3. Chemical Space Analysis and Grouping

The MoViz pipeline [67] was employed for chemical space analysis and grouping. The NICEATM team developed the pipeline, which facilitates identifying and organizing structurally similar compounds. Morgan fingerprints with a radius of 2 and 2048 bits were used for molecular descriptor calculation during the analysis. After this, low-variance descriptors were filtered out, and variable selection was performed to enhance the robustness of the analysis. Subsequently, uniform manifold approximation and projection (UMAP) [68] was utilized for dimensionality reduction, enabling the visualization of complex chemical spaces in a simplified manner. UMAP was chosen over linear methods like principal component analysis (PCA) because it is a nonlinear manifold learning technique that can better preserve both local and global structures of high-dimensional data, and it also involves no computational restrictions on embedding dimensions, which is advantageous for handling the high-dimensionality molecular descriptors used [68,69]. The MoViz [67] pipeline allowed for the effective grouping of compounds, supporting the identification of structural similarities and potential activity cliffs.

### 2.4. Activity Cliff Analysis—Molecular Roughness Calculation

Mordred descriptors were normalized to compute a pairwise distance matrix based on Euclidean distance. The roughness index (ROGI) score was calculated to quantify the ruggedness of the activity landscape [70]. For visualization, we projected the high-dimensional data onto a 2D plane using multidimensional scaling (MDS). The 3D plot was generated by interpolating the activity values across the projected 2D coordinates and plotting the resulting surface. Additionally, a 2D contour plot was created to highlight the activity distribution, utilizing interpolation to create a smooth heatmap overlaying the 2D MDS projection. These visualizations facilitated the identification of activity cliffs, providing insights into structure–activity relationships within the continuous data set. The code for these plots was adapted from https://github.com/coleygroup/rogi-results, accessed on 18 October 2024 [70].

### 2.5. Case Study: Validation Using Defined Approaches

Our models were validated using compound results from the NIEHS Defined Approaches for Skin Sensitization Web app (DASS app), representing testing strategies that are accepted by international regulatory authorities. The original DASS data set contained 196 compounds. These compounds cover a diverse range of chemical structures and sensitization potencies and have been employed in previous studies to assess the performance of the novel skin sensitization-defined approaches, as recognized by the OECD TG 497 and accepted in 2021 [5]. The data extracted from the DASS app are also available in the National Toxicology Program (NTP) Integrated Chemical Environment (ICE), a highly curated repository of toxicological data for various endpoints [71,72]. We removed compounds with inconclusive outcomes and compounds in our model’s training set. Then, we used an automated QSAR-ready workflow to standardize and curate chemical structures [73]. For binary models, we performed validation using (i) the Defined Approaches Integrated Testing Strategy (DA ITS) Call, (ii) 2o3 Defined Approaches Call, and (iii) Basketter Human data Call as references [74,75]. After the curation protocols, the DA ITS call had 138 compounds (38 NC, 100 sensitizers), the 2o3 Call had 142 compounds (61 NC, 81 sensitizers), and the Basketter data set had 63 compounds (21 NC, 42 sensitizers). A consensus heatmap was created using the Seaborn [76] package for the 45 compounds that had predictions for all three validation data sets for comparison.

The “Basketter Potency” and “HPPT Reference Potency” columns were used to validate multiclass models. After the removal of training, unclassified, and automated curation, the data sets comprised 81 compounds (13 NC, 27 weak sensitizers, and 14 strong sensitizers) and 14 compounds (2 NCs, 10 weak sensitizers, and 2 strong sensitizers), respectively.

The Y-randomization test was also used per OECD Guidance Document on the validation of QSAR models [77] to investigate whether the reported accuracy of our models was due to chance correlation. The results of ten rounds of Y-randomization are reported in Table S3.

## 3. Results and Discussion

### 3.1. Data Sets Summary

Here, we have collected and curated the HPPT and the HPPT Classifications database into four independent binary classification data sets for modeling and comparison, four multiclass data sets, and three continuous data sets. Table 2 demonstrates the number of compounds in each data set used for binary classification model development. Data curation is essential for the development of QSAR models [29,40]. During curation, we found various inconsistencies in which the same compound would possess the conflicting ranking of both a sensitizer and a non-sensitizer; these entries were removed. We also identified multiple entries of the same compound with concordant outcomes, and these were merged into a single entry. We emphasize the need for rigorous biological curation, as QSAR models developed with duplicate entries will exhibit under-optimistic performance if the outcomes are dissimilar or over-optimistic performance if the outcomes are identical. The number of compounds in the multiclass data set (Table 3) and continuous models (Table 4) are provided below. We note that there was a class imbalance for the binary and multiclass data sets; sensitizers were more abundant than non-sensitizers. Weak sensitizers were roughly three times as abundant for multiclass data as other classes. The majority class was randomly undersampled in the multiclass models to mitigate this imbalance and improve model performance.

### 3.2. Binary Classification QSAR Models

Here, we built 36 binary classification models with different combinations of molecular fingerprints and ML methods. The RF, LightGBM, and SVM models were built using Python and validated with 5-fold cross-validation. Table 5 highlights the statistical characteristics of the binary classification models after threshold moving (calibrated). Table S1 highlights the characteristics of the uncalibrated models. Threshold moving was used to increase prediction confidence without losing data (i.e., we attempted a threshold-moving calibration of probability estimates without the need to balance the data). Most cross-validated skin sensitization models showed high-quality predictive accuracy on 5-fold external cross-validation based on metrics such as CCR, SE, SP, PPV, NPV, and coverage.

Table 5 highlights the best-performing model for each data set. Briefly, the performance of these four models showed reasonable CCR (74–88%), SE (63–92%), PPV (90–97%), NPV (41–60%), and AUC (75–92%). The best-performing model, using the WES data set with ECFP4 and SVM, showed the most promise and was deployed as a web tool. The performance of all models in the WES data set was also assessed using the Y-randomization test. The best-performing WES model, with the ECFP4 and SVM, significantly outperformed models built with Y-randomization (Table S3).

#### 3.2.1. Threshold Moving

**Figure 2:**
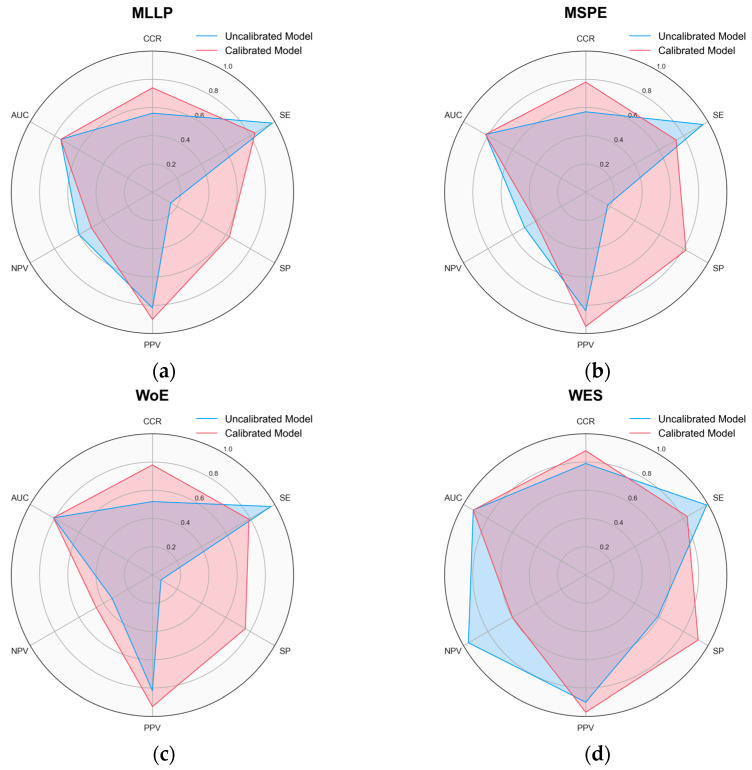
Radar chart comparing metrics before and after calibration for best-performing models. CCR, SE, SP, PPV, NPV, and AUC are included. AUC is a threshold-independent metric. The results for the best-performing (**a**) MLLP, (**b**) MSPE, (**c**) WoE, and (**d**) WES models. The calibrated models outperformed the uncalibrated models or scored similarly, rarely underperforming models without calibration. Generally, a more balanced and symmetrical shape in the radar chart indicates a uniform performance across the metrics, while pronounced peaks and dips highlight potential strengths and weaknesses of the models, respectively.

In the developed RF, SVM, and LightGBM models, a continuous value represents the probability of a given compound belonging to a specific class (i.e., sensitizer or non-sensitizer). With the aim of better differentiating between skin sensitization sensitizers and non-sensitizers, despite the imbalance of classes present in the data, the probability threshold was adjusted. Usually, probabilities less than 0.5 are assigned to the non-sensitizing class (Class 0), while values greater than or equal to 0.5 are assigned to the sensitizing class (Class 1). However, when modeling imbalanced data, QSAR models often express lower probability estimates for the minority class [49]. Thus, we tested various probability thresholds ranging from 0 to 1 in order to identify the optimal threshold for model performance. The threshold-moving approach and calibration significantly improve the statistical performance of the QSAR models. Figure 2a–d highlights that, for the best models, the calibrated models outperformed the uncalibrated counterparts or scored similarly, rarely underperforming models without calibration. Table S1 highlights the uncalibrated performance for all binary models.

#### 3.2.2. Binary Model Interpretation with SHAP

**Figure 3:**
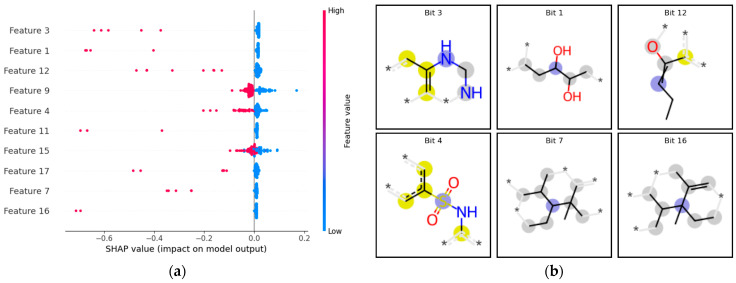
SHAP interpretation of the best ECFP4 binary models. (**a**) MLLP model: The x-axis indicates SHAP values and the impact of molecular bits on model output; the y-axis represents compound features (bits). Red denotes a positive effect on model impact; blue represents a negative impact on model prediction. (**b**) The highest impact features on model performance are highlighted. The blue contour atoms are central atoms in the feature; yellow represents aromatic atoms, and gray represents aliphatic ring atoms.

SHAP values were calculated to interpret the built binary classification ML models. SHAP calculates the model’s feature importance, and it is accessible in the web tool. Figure 3a shows the most relevant features, ranked in order of the impact on the model’s predictions. These features exhibited the highest average absolute SHAP values, suggesting that they strongly influenced the model’s predictions. Figure 3b demonstrates the bit images of the highest impact features. Bits 7 and 16 shared similar substructures, indicating that this substructure is likely important for the model’s predictions. Given the imbalance in the data set, most high-impact bits showed a higher impact on the prediction of sensitizers. It appears that bits 9 and 15 had the highest impact on non-sensitizer prediction. Since a chemical fingerprint of radius 2 was used, many of the substructures highlighted lack robust evidence of chemical significance. However, comparisons can still be drawn for some substructures. For instance, bit 4 resembles the sulfonamide moiety, and drugs with this structural composition have reported common adverse drug reactions such as allergies and hypersensitivity [78,79,80,81,82]. According to the Adverse Outcome Pathway (AOP) for skin sensitization by the OECD [83], step 2 of the AOP involves the substance behaving as a direct-acting electrophile or being converted into a reactive electrophilic metabolite. Given the electrophilic nature of the sulfonamide moiety, as discussed in a recent article evaluating the degradation mechanisms of sulfonamides [84], it is plausible that this structural feature could interact with nucleophilic sites in proteins, potentially contributing to sensitization processes, as outlined in the AOP for skin sensitization. Further, bit 1 resembles a 1,2 alkane diol substructure, and links have been examined between changes in 1,2 alkane diol chain length and skin sensitization potential [85].

#### 3.2.3. Case Study: Validation of Binary Models with DASS App Defined Approaches

Using the DASS app reference experimental values, we compared our binary model’s predictions to the OECD-validated defined approaches for assessing skin sensitization (Table 6), as well as a literature-based human reference set.

The model showed comparable performance across all three validation scenarios, with accuracy ranging from 61 to 75% and SE from 88 to 94%. Notably, the model showed significant strength in predicting sensitizers (high SE and PPV) compared to non-sensitizers (low SP and reasonable NPV). Predictions for the Basketter data set showed promise, which was expected since the Basketter data most closely resemble the model’s training data, given that it expresses expert judgment based on human testing, while the DA ITS Call and the 2o3 are based on an in vitro screening of compounds.

Figure S1 highlights the discrepancy between the predictions for each reference, highlighting how the data used for validation can impact the validation metrics. In brief terms, compounds with all reference values concordant across the three data sets (i.e., all three columns are either “1” or “0”) showed high correct prediction rates, correctly labeling 30 out of 36 (83%) compounds. When the original outcomes of the external values were divergent, such as in compounds of index 1, 5, 40, and 44, the model usually predicted the Basketter category correctly but incorrectly according to the DA ITS and 2o3 Call. Such discrepancies were anticipated, considering that the model was created using human data, and the Basketter call is also based on human information, whereas the DA ITS Call and the 2o3 Call are based primarily on in vitro experiments (DPRA, h-CLAT, and KeratinoSens assays).

### 3.3. Chemical Space Analysis

**Figure 4:**
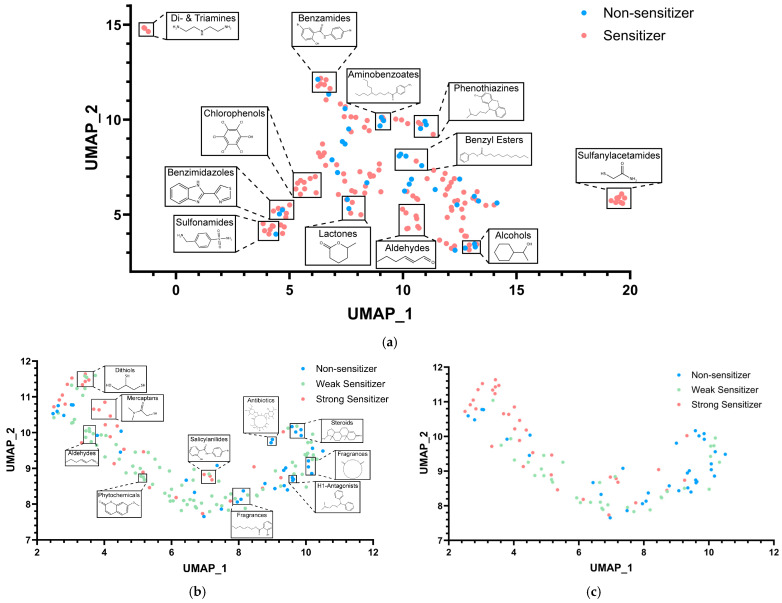
Supervised classification results. Compounds are represented as points on the X and Y axes. The chemical grouping of compounds in the data set was performed after using Morgan descriptors with a radius of 2 and 2048 bits. Low-variance descriptors were filtered, and dimensionality reduction was performed using SVM. Grouping was performed after the calculating was depicted for each of the data sets. Each cluster can be identified by different colors in the chart. If the user downloads the data set and utilizes the pipeline shown in MoViz [67], the option to interact with points in the plot and visualize the chemical structures is available. Here shown are (**a**) clustering for the WES data set, (**b**) clustering for WES multiclass data, and (**c**) clustering for the WES data set after random balancing. Non-sensitizers are in blue, weak sensitizers are in green, and strong sensitizers are in red.

The chemical space analysis performed was investigated for each of the best-performing models. Supervised classification was performed to identify similarities and gaps in the chemical space. Figure 4a highlights that there is not much overlap between classes (i.e., structurally similar compounds but with different outcomes). Despite variable selection, compounds were still aggregating and clustering due to structural similarities. Here, we classified compounds that appeared to cluster together to identify the chemical space, and we found clusters of chlorophenols, sulfanylacetamides, di-amines, and tri-amines to contain mostly skin sensitizers.

A similar process was conducted for the best-performing multiclass data set (Figure 4b,c). The multiclass also had a highly diverse set of compounds in its chemical space. After random undersampling, Figure 4c highlights that the model still covers most of the chemical space. Random undersampling was beneficial in balancing class distribution by reducing the size of the majority class while mitigating the risk of bias in our ML models. In this situation, random undersampling yielded a reasonable alternative, as most compounds of the majority class (weak sensitizers) were structurally similar and in close proximity to one another (Figure 4b).

### 3.4. Multiclass Modeling Results

Table 7 demonstrates the metrics for the best-performing multiclass models developed. The performance of all built multiclass models can be found in Table S1. The WES data set showed the most promising results: reasonable accuracy (73%), specificity (82%), and an AUC of 75% with 81% coverage. Compared to the binary models, the multiclass models surprisingly showed better classification of negatives than positives (high SP and NPV). One potential explanation is the balancing of the data sets and the relatively small number of compounds in the data set, which likely contributed to better discrimination between strong sensitizers and non-sensitizers. The best-performing multiclass model also performed better than the models built with Y-randomization (Table S3).

#### Case Study: Validation of Multiclass Models with Defined Approaches

**Figure 5:**
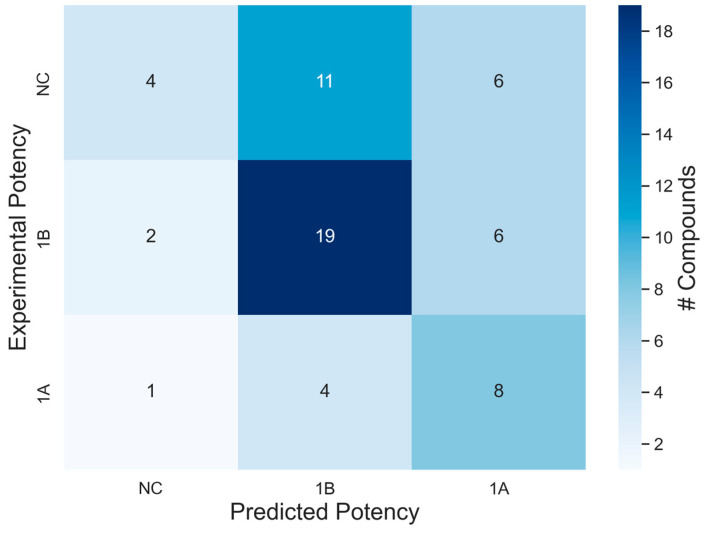
Confusion matrix for multiclass model classification validation results using DA Basketter data. Non-sensitizers were the class with the most incorrect predictions. Color coding represents the number of compounds; the classes are NCs—non-sensitizers; 1B: weak sensitizer; 1A strong sensitizer.

The validation performance of the multiclass models was evaluated using human skin sensitization potency reference values as published by the OECD. Here, we can observe that the model exhibits an average prediction accuracy of (51%), correctly predicting 30 of the 58 compounds. As the confusion matrix highlights (Figure 5), the multiclass model showed good distinctions between the non-sensitizers (NCs) and strong sensitizers (1A), only incorrectly mislabeling one compound as a strong sensitizer. However, as emphasized in Figure 4, the model struggled to correctly distinguish between weak and non-sensitizers due to their structural similarity (Figure 4), so most NCs were incorrectly predicted as weak sensitizers (11 compounds). The non-sensitizing class had the most incorrect labels (17 out of 21 compounds), likely due to the lack of true negatives during model building and structural similarity to weak sensitizers. Table S2 contains each compound’s predictions and the validation predictions using HPPT data. For the external set of HPPT compounds in the DASS app, 13 of 14 compounds were inside the applicability domain. The model correctly predicted 9 of the 13 compounds in the HPPT validation set (69%). Overall, when tested with compounds outside the model’s training set, the multiclass model showed reasonable promise in predicting human skin sensitization potential, with average class-wise CCR of 63%, SP of 75%, SE of 51%, PPV of 51%, and NPV of 76%.

The validation data set included a diverse array of compounds representing various chemical classes, such as aldehydes, alcohols, esters, acids, amines, phenols, and hydrocarbons (Table S2). The original data in the DASS app are also available in the NTP’s Integrated Chemical Environment (ICE) [72,86]. In the ICE toolbox, the data set is accessible via the Chemicals quick list as “OECD Defined Approach to Skin Sensitization: Human” (https://ice.ntp.niehs.nih.gov/ChemicalQuickLists, accessed on 18 October 2024). The ICE toolbox Chemical Characterization workflow can also be used to enable a better understanding of the chemical composition of the data set.

### 3.5. Visualization of Continuous Data Structure–Property Landscapes

**Figure 6:**
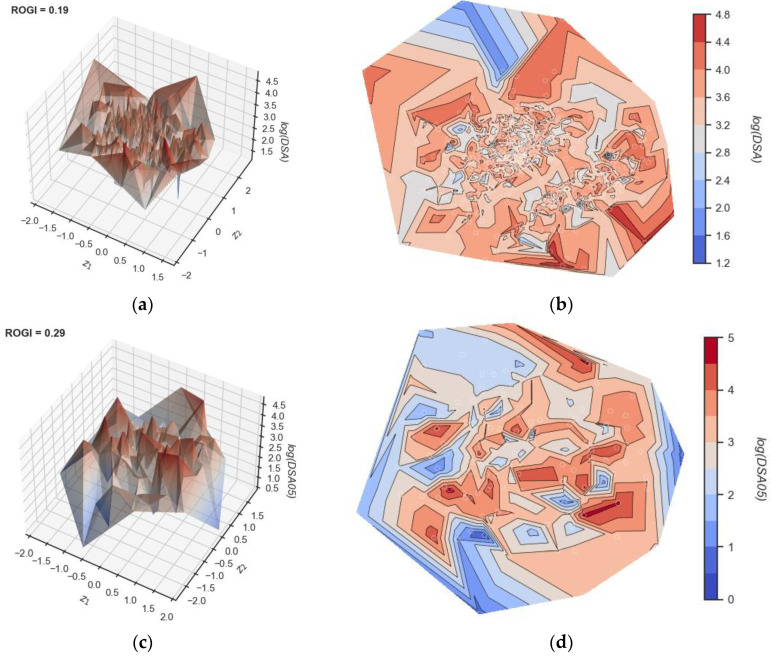

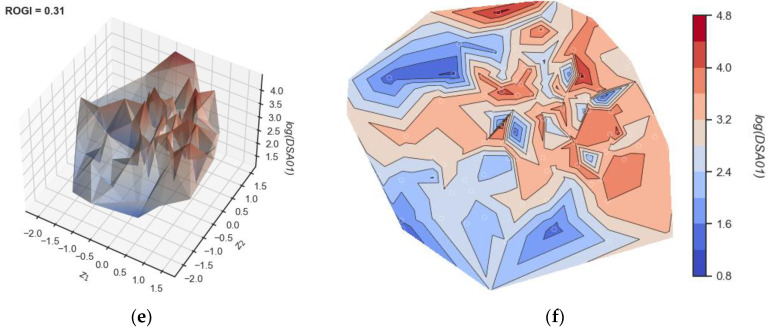
Visualization of molecular landscapes of the different data sets. After Euclidean distance calculation, the three high-dimensionality datasets are projected onto a 2D plane (coordinates z1 and z2). The third dimension corresponds to the activity value of each data set (log values of DSA, DSA05, or DSA01). Mordred descriptors were used to eliminate recursive features. (**a**,**c**,**e**) corresponds to the 3D representation, while (**b**,**d**,**f**) correspond to the two-dimensional contour plots. (**a**,**b**) log(DSA), (**c**,**d**) log(DSA05), and (**e**,**f**) log(DSA01). The ROGI value for each data set is shown. Activity cliffs are visible as areas in proximity with highly different activity values.

Visualizing the continuous skin sensitization data provides a qualitative representation of molecular roughness. Through the 3D and 2D landscapes, activity cliffs can be easily identified. The data in Figure 6a,b for the DSA data comprised the smoothest landscape of the three, with a ROGI value of 0.19, followed by DSA05 at 0.29 (Figure 6c,d) and then DSA01 at 0.31 (Figure 6e,f). In Figure 6, we can observe that all landscape profiles exhibit a relatively rugged landscape throughout. Further, for the three data sets, we can observe that very similar molecules lead to drastically different outcomes, suggesting that these data sets contain activity cliffs.

The continuous models developed for predicting human skin sensitization did not perform satisfactorily. The box and whisker plots (Figure S2) highlight one of the challenges in modeling human HPPT data due to the high variability and wide range of results for a single chemical that, when expanded to the entire data set, introduces noise and inhibits the model’s ability to predict the dose per skin area for compounds correctly. Notably, for the three continuous data sets (Figure S2a–c), most compounds have a wide range and interquartile range (IQR), suggesting that using these compounds for modeling would introduce ambiguity and significant uncertainties into the model’s predictions. These results underscore the limitations of using continuous models to capture the underlying patterns in the human predictive patch test data. We recommend that other groups build on our curated continuous data and explore alternative modeling approaches to achieve more reliable predictions.

### 3.6. Model Implementation

**Figure 7:**
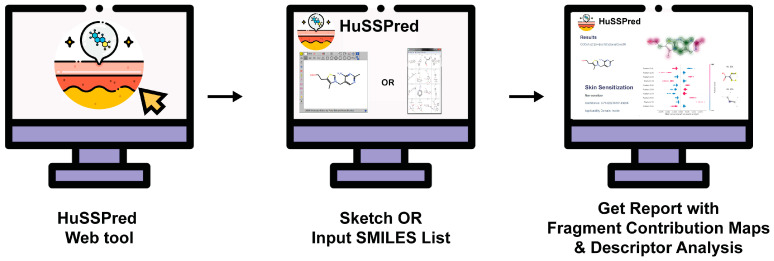
Model implementation and user-friendly pipeline of HuSSPred models.

The most predictive classification model for Human Skin Sensitization Prediction was implemented in the open-access HuSSPred web application (https://husspred.mml.unc.edu, accessed on 18 October 2024). The HuSSPred web tool was designed to possess an intuitive user interface, where users can draw compounds of interest in the “molecular editor” box or directly paste a list of SMILES strings to query chemical compounds (Figure 7). After clicking the “Get Properties” button, the user will be shown the classification outcome of the compounds (sensitizer or non-sensitizer) using the best classification model. Further, the user can opt to include ‘Fragment contribution analysis” and “SHAP descriptor importance” in the report. All predictions also contain AD estimates and mechanistic interpretation using color-coded maps of fragment contribution. For the fragment-contribution maps, atoms or fragments promoting positive toxicity are highlighted in green, while those decreasing toxicity are highlighted in purple. The models developed in this study are available within the HuSSPred web application https://husspred.mml.unc.edu/, accessed on 18 October 2024).

In the development of NAMs to predict a compound’s toxicity and sensitization potential, it is essential for models to accurately predict potential skin sensitizers, rather than non-sensitizers, which can reduce animal testing and the waste of resources by eliminating compounds likely to fail downstream in the development process. Given these safety and resource management considerations, the high sensitivity of the best-performing models (63–92%) and high PPV (90–97%) indicate that sensitizers are frequently correctly labeled. Further, the high PPV and reasonable NPV underscore the model’s potential to contribute to advancing the three Rs (reduce, refine, and replace). The model’s applicability to binary contexts is further supported by the high sensitivity (94%) and accuracy (75%) obtained during the model’s validation with reference human data and other human biology-based DAs for skin sensitization testing.

While the model performance could be drastically improved by including a more comprehensive set of true negatives, we emphasize the difficulty of obtaining such, given the current human skin sensitization testing system. Currently, the Globally Harmonized System of Classification and Labelling of Chemicals (GHS) [74,87] uses a set of frameworks for classifying compounds as skin sensitizers [88]. Therefore, the challenge of obtaining a true negative is commonly caused by not testing substances at a high enough concentration to trigger sensitization. This frequently results in compounds labeled as “inconclusive”, which are unsuitable for modeling. To our knowledge, this skin sensitization QSAR model’s high sensitivity fares competitively well against other established tools in the field. An evaluation of in silico tools to predict skin sensitization was published in 2017 [89]. From this report, the best-performing and best-known tools were VEGA, Derek Nexus, and the OECD QSAR Toolbox. According to the metrics reported in the assessment, our tool exhibited a good external validation sensitivity score relative to those three, with 94% for the Basketter human data set, compared to 91%, 78%, and 43%, respectively for the other three tools, while also being one of few developed with human data. For other metrics, such as CCR, SP, PPV, and NPV, our models performed similarly or better in the majority of cases.

Here, we suggest that our skin sensitization QSAR models’ utility lies in their ability to predict skin-sensitizing substances accurately. The freely available web tool HuSSPred was built on the best-performing models with the most highly curated data set available, making it a valuable tool for toxicity prediction and providing risk assessors with confidence in the model’s positive predictions. The overall weight-of-evidence approach used for the web tool (WES) models was the preferred data set due to its robust scoring system and overall performance. The scores assigned to each “extrapolated” classification in the WES database intuitively reflect how a risk assessor might combine multiple HPPT results in a single WoE assessment. Only unambiguous outcomes were reported and assigned a score, so the developed models available in the web tool were built only on the most rigorous HPPT data [27,28].

There is an increasing need to quickly determine a compound’s skin sensitization potential from a regulatory standpoint; unfortunately, these studies are time-consuming and expensive, and they raise ethical concerns. On the other hand, our QSAR models can be easily implemented in the early stages of drug development and testing, minimizing resource waste and facilitating the early stages of drug development while reducing animal testing. We hope the QSAR models developed here progress further towards regulatory acceptance.

## 4. Conclusions

Overall, we have carefully curated the HPPT database to predict skin sensitization, enabling a more accurate assessment of the human response. The models underwent rigorous validation with reference data from the internationally validated Defined Approaches for Skin Sensitization (DASS), resulting in one of the first in silico tools based strictly on human data for skin sensitization, with high accuracy and sensitivity, as validated with human biology-based in vitro outcomes and human data. The rigorously curated data, validation predictions, and models are available on the website (https://husspred.mml.unc.edu/, accessed on 18 October 2024), where users can select the desired models to obtain (i) binary predictions, (ii) multiclass potency predictions for hazard assessments of compounds, and (iii) fragment-contribution maps for each assay prediction.

## Figures and Tables

**Table 1:** Overview of existing skin sensitization prediction software.

Title	Computational Approach	Species/Assay	Access
StopTox [12]	QSAR	Mice/LLNA	Free
PredSkin [13]	QSAR	Mice/LLNA	Free
CASE Ultra [14]	Structural alerts	Mice/LLNA	Commercial
Derex Nexus [15,16]	Structural alerts Expert system	Mice/LLNA	Commercial
OECD QSAR Toolbox [17]	QSAR and read-across	- *	Free
VEGA [18]	Read-across	-	Free
ToxTree [19]	Structural alerts	-	Free
TOPKAT [15]	QSAR	Rat & Other/ Multiple	Commercial

* Cells without input indicate that this information is not publicly available or multiple models are encompassed in the tool, making a single answer not applicable.

**Table 2:** Number of compounds in each binary data set for MSPE, MLLP, WoE, and WES classifications.

Data Set	Sensitizers	Non-Sensitizers	Total
MLLP	154	41	195
MSPE	177	39	216
WoE	177	41	218
WES	132	37	169

MLLP, median location-like parameter; MSPE, median sensitization potency estimate; WoE, individual weight-of-evidence scores; WES, overall weight-of-evidence scores.

**Table 3:** Number of compounds in each multiclass data set for MSPE, MLLP, WoE, and WES classifications.

Data Set	Strong Sensitizers	Weak Sensitizers	Non-Sensitizers	Total
MLLP	40	118	41	199
MSPE	44	123	39	206
WoE	42	126	41	209
WES	37	87	34	158

MLLP, Median location-like parameter; MSPE, median sensitization potency estimate; WoE, individual weight-of-evidence scores; WES, overall weight-of-evidence scores.

**Table 4:** Number of compounds in continuous data sets for DSA, DSA05, and DSA01 classifications.

Data Set	Total
DSA	829
DSA05	170
DSA01	104

DSA, dose per skin area; DSA05, dose per skin area for sensitization of 5% of the tested population; DSA01, dose per skin area for sensitization of at least 1 tested individual.

**Table 5:** Calculated metrics for calibrated binary classification QSAR models.

Approach	FP	ML	CCR	SE	SP	PPV	NPV	AUC	Coverage	PT
MLLP	**ECFP4**	**RF**	**0.74**	**0.84**	**0.63**	**0.90**	**0.50**	**0.75**	**83**	**0.77**
	ECFP4	SVM	0.71	0.81	0.61	0.89	0.45	0.77	83	0.78
	ECFP4	LightGBM	0.62	0.55	0.68	0.88	0.27	0.63	83	0.80
	Mordred	RF	0.76	0.69	0.83	0.94	0.40	0.80	93	0.78
	Mordred	SVM	0.62	0.83	0.41	0.85	0.38	0.58	93	0.80
	Mordred	LightGBM	0.74	0.69	0.78	0.93	0.39	0.74	93	0.82
MSPE	ECFP4	RF	0.72	0.85	0.59	0.90	0.47	0.72	88.9	0.80
	ECFP4	SVM	0.68	0.75	0.62	0.90	0.35	0.73	88.9	0.84
	ECFP4	LightGBM	0.55	0.60	0.51	0.85	0.22	0.56	88.9	0.82
	**Mordred**	**RF**	**0.78**	**0.74**	**0.82**	**0.95**	**0.41**	**0.82**	**94**	**0.77**
	Mordred	SVM	0.70	0.67	0.72	0.91	0.32	0.74	94	0.85
	Mordred	LightGBM	0.73	0.67	0.79	0.94	0.35	0.77	94	0.82
WoE	ECFP4	RF	0.74	0.75	0.73	0.92	0.40	0.75	88.5	0.83
	ECFP4	SVM	0.72	0.88	0.56	0.90	0.52	0.70	88.5	0.78
	ECFP4	LightGBM	0.62	0.49	0.76	0.90	0.25	0.58	88.5	0.81
	**Mordred**	**RF**	**0.78**	**0.79**	**0.76**	**0.93**	**0.46**	**0.81**	**92**	**0.73**
	Mordred	SVM	0.56	0.64	0.49	0.84	0.24	0.53	92	0.81
	Mordred	LightGBM	0.74	0.72	0.76	0.93	0.39	0.75	92	0.81
WES	ECFP4	RF	0.82	0.89	0.76	0.93	0.65	0.88	90.5	0.75
	**ECFP4**	**SVM**	**0.88**	**0.83**	**0.92**	**0.97**	**0.60**	**0.92**	**90.5**	**0.84**
	ECFP4	LightGBM	0.64	0.53	0.76	0.89	0.31	0.60	90.5	0.78
	Mordred	RF	0.73	0.67	0.78	0.92	0.4	0.79	91	0.77
	Mordred	SVM	0.6	0.71	0.49	0.83	0.32	0.59	91	0.78
	Mordred	LightGBM	0.74	0.79	0.68	0.9	0.47	0.74	91	0.74

RF, random forest; ECFP4, extended connectivity fingerprints with diameter 4; LightGBM, light gradient-boosting machine; MACCS, molecular access systems keys fingerprint; CCR, correct classification rate; SE, sensitivity; SP, specificity; PPV, positive predictive value; NPV, negative predictive value; coverage, a ratio of the test set or external set compounds within the applicability domain; PT, probability threshold; statistical results all obtained after threshold-moving calibration. Statistical results obtained from the default probability thresholds (uncalibrated) are available in the Supplementary Table S1. Models in bold reflect those with the best performance in the data set.

**Table 6:** Case study validation metrics for binary classification QSAR models.

Reference Column	# Compounds (NCs/Sensitizers)	Inside AD	ACC *	SE *	SP *	PPV *	NPV *
DA ITS call	138 (37/101)	115	61%	88%	34%	75%	54%
2o3 DA call	142 (61/81)	117	62%	88%	28%	61%	64%
Basketter human data	63 (21/42)	48	75%	94%	34%	75%	75%

* Metrics reflect predictions inside the model’s applicability domain.

**Table 7:** Calculated metrics for calibrated multiclass QSAR models with undersampling.

Approach *	FP	ML	CCR	SE	SP	PPV	NPV	AUC	Coverage
WES	ECFP4	RF	0.60	0.47	0.74	0.49	0.73	0.69	49
	ECFP4	SVM	0.58	0.44	0.72	0.44	0.72	0.66	49
	ECFP4	LightGBM	0.56	0.41	0.71	0.42	0.71	0.61	49
	**Mordred**	**RF**	**0.73**	**0.64**	**0.82**	**0.64**	**0.82**	**0.75**	**81**
	Mordred	SVM	0.50	0.34	0.67	0.23	0.67	0.48	81
	Mordred	LightGBM	0.66	0.55	0.77	0.55	0.78	0.69	81

* Metrics for the remaining approaches are available in Table S1.

## Data Availability

The data presented in this study are available at https://github.com/molecularmodelinglab/, accessed on 18 October 2024.

## Supplementary Materials

The following supporting information can be downloaded at https://www.mdpi.com/article/10.3390/toxics12110803/s1. File S1: Curated data sets for binary, multiclass, and continuous model development. Table S1: Uncalibrated model results for binary and WoE approaches for multiclass models. Table S2: Validation exercise data sets and predictions. Table S3: Y-randomization test modeling results. Figure S1: Model predictions for compounds in common across validation datasets. Figure S2: Box and whisker plots for continuous data.
