# Supplementary material for: A Novel Machine Learning Model and a Web Portal for Predicting the Human Skin Sensitization Effects of Chemical Agents
Source: Toxics. 2024 Nov 7;12(11):803. doi: 10.3390/toxics12110803 (PMC11598222; doi:10.3390/toxics12110803)
Supplement: Supplementary file 1 [file toxics-12-00803-s001.zip › Supplementary Figures.pdf]

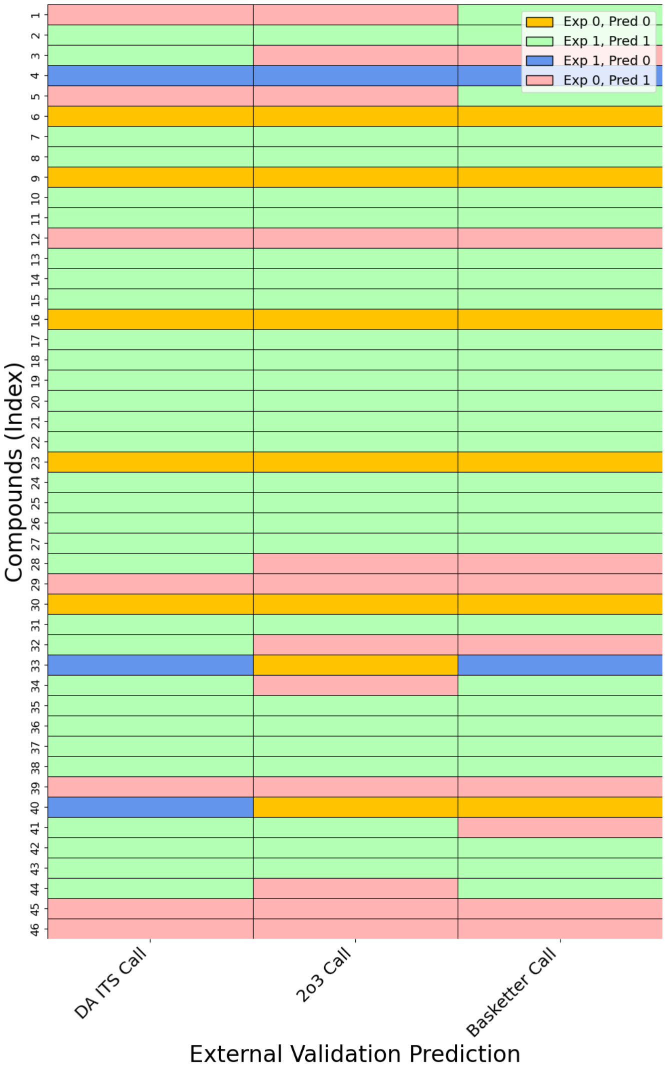

Figure S1. Case study predictions for compounds present in all three validation sets. Compound indexes are available (see S3 for detailed compound information). The model's predictions are benchmarked against the experimental values. Predictions are color-coded as follows: true negatives (*in yellow*), true positives (*in green*), false negatives (*in blue*), and false positives (*in red*). The figure demonstrates that, when outcomes were concordant across all three validation calls, the model accurately predicted 30 of 36 outcomes. When these external validation experimental outcomes differed, the model frequently predicted results according to the human data present in the Basketter call.

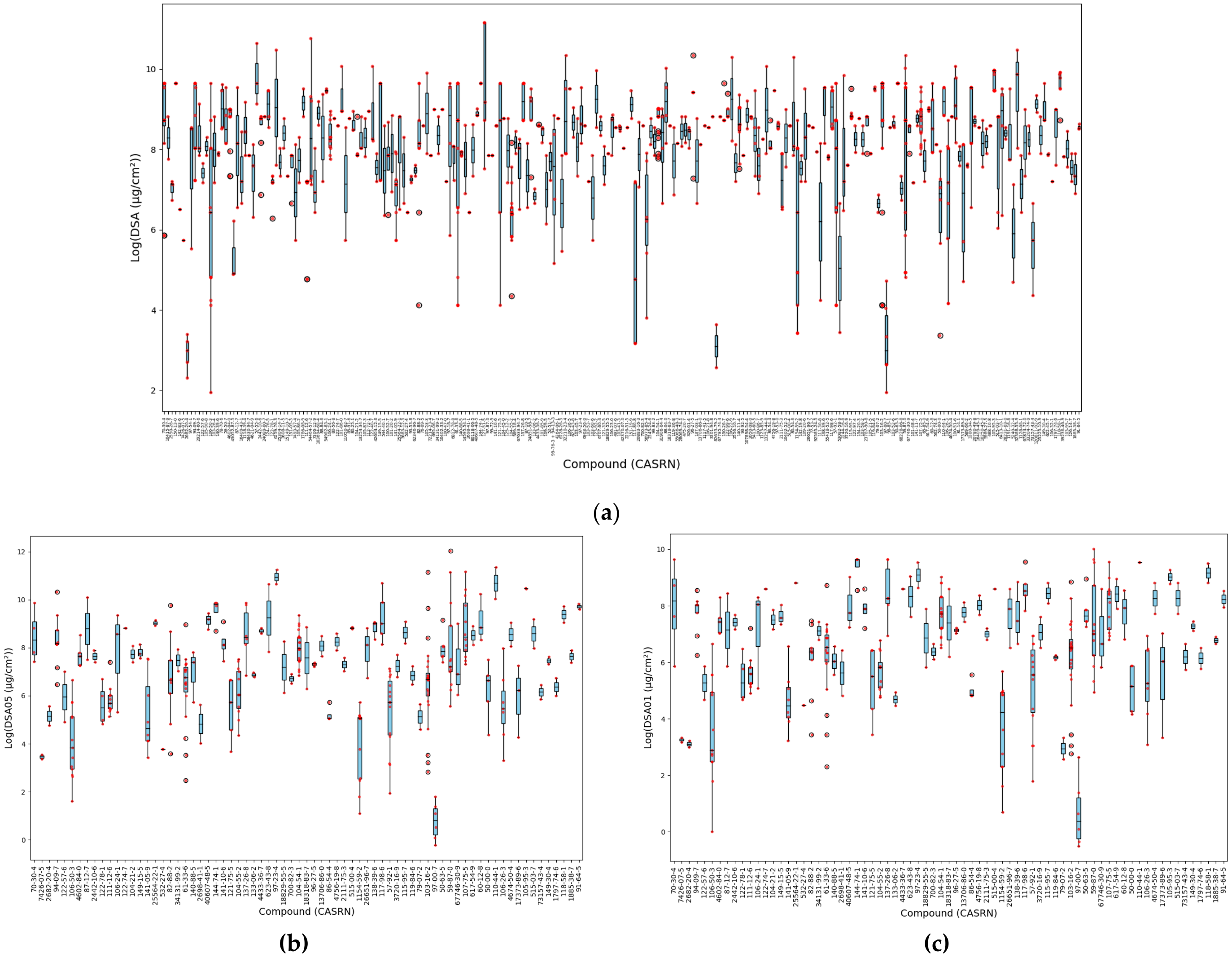

Figure S2. Box and whisker plots for continuous data. Red indicators represent compounds; whiskers demonstrate the interquartile range. The CASRN of the compounds is provided on the x-axis, and the log of the activity is shown on the y-axis. (**a**) DSA data set. (**b**) DSA05 data set. (**c**) DSA01 data set.
